# A Pair of Coupled Waveguides as a Classical Analogue for a Solid-State Qubit

**DOI:** 10.3390/s22218286

**Published:** 2022-10-28

**Authors:** Andrey E. Schegolev, Nikolay V. Klenov, Anna V. Bogatskaya, Rustam D. Yusupov, Alexander M. Popov

**Affiliations:** 1 D. V. Skobeltsyn Institute of Nuclear Physics, Moscow State University, 119991 Moscow, Russia; 2 Science and Research Department, Moscow Technical University of Communication and Informatics, 111024 Moscow, Russia; 3 Faculty of Physics, Moscow State University, 119991 Moscow, Russia; 4 P. N. Lebedev Physical Institute, Russian Academy of Sciences, 119991 Moscow, Russia

**Keywords:** integrated photonics, slow-varying amplitude approximation, Schrödinger equation, double-well potential, qubit, neuron

## Abstract

We have determined conditions when a pair of coupled waveguides, a common element for integrated room-temperature photonics, can act as a qubit based on a system with a double-well potential. Moreover, we have used slow-varying amplitude approximation (SVA) for the “classical” wave equation to study the propagation of electromagnetic beams in a couple of dielectric waveguides both analytically and numerically. As a part of an extension of the optical-mechanical analogy, we have considered examples of “quantum operations” on the electromagnetic wave state in a pair of waveguides. Furthermore, we have provided examples of “quantum-mechanical” calculations of nonlinear transfer functions for the implementation of the considered element in optical neural networks.

## 1. Introduction

Computing (including quantum computing) devices based on integrated photonics (IP) are in active use nowadays in analog-to-digital converters, optical neural networks, boson sampling systems, etc. [1,2,3,4,5,6]. However, linear operations and data transfer are still the main tools on this platform. In particular, in the field of quantum computing, the most efficient control of qubit states has been demonstrated for solid-state qubits. In the context of this work, we would like to highlight promising implementations of quantum bits (qubits) which are based on the general features of the energy spectrum of the double-well potential [7,8,9,10,11,12,13,14,15,16]. In various flux qubits and double quantum dots, tunneling splitting of the ground state allows the formation of a qubit basis. The control of the barrier and the asymmetry of the potential allows one to implement complete sets of qubit operations.

Therefore, the development of IP-elements with a nonlinear transformation of the input signal is in high demand. Here, it is necessary to mention a neuron with a given (in particular, a sigmoidal) transfer function for optical neuro nets [6]. Another example of an element with a significantly nonlinear response is an optical analog of an artificial two-level quantum system.

In this article, we propose a new approach to analyze the elements based on one of the simplest IP-structures—a pair of relatively close-spaced planar waveguides. In the framework of the optical-mechanical analogy [17], this system of waveguides corresponds to a double-well potential where the potential energy plays the role of susceptibility χ(x). In our case, by selecting (adjusting) the values of the permittivity for waveguides and the spacer between them, one can realize various transformations for the incoming radiation for the given spatial dimensions of the structure. In other words, it is possible to implement various specified transfer functions for a simple IP-element. Both the classical optical analog of the solid-state qubit and the optical neuron can be implemented on this basis (using only room-temperature sensors).

In Section 2, we present a description of the apparatus used in the framework of the optical-mechanical analogy for modeling the operations with the proposed nonlinear element. In Section 3, we provide a numerical analysis of various operation modes of the proposed “classical optical analogue” of a double quantum dot or a flux qubit. Finally, we will discuss challenges and opportunities associated with the implementation of the developed concept for further applications in integrated photonics circuits.

## 2. Model and Methods

### 2.1. Optical-Mechanical Analogy and General Scheme of the Optical Nonlinear Element

In this article, we consider the wave equation for light propagation in an inhomogeneous medium

(1) $$\nabla^{2}\vec{E}=\frac{n_{0}^{2}}{c^{2}}\frac{\partial^{2}\vec{E}}{\partial t^{2}}+\frac{4\pi \chi (\vec{r})}{c^{2}}\frac{\partial^{2}\vec{E}}{\partial t^{2}},$$

here $\vec{E}$ is the electric field strength, $n_{0}=\sqrt{\varepsilon }$ is the refractive index of the medium and ε is its permittivity that are assumed to be constant over the space; $\chi (\vec{r})$ is the part of susceptibility that depends on the spatial coordinates. If $\left|\nabla \chi \right|<<\left|\nabla E\right|/\left|E\right|$, i.e., the susceptibility is a slow-varying function in space, the slow-varying amplitude (SVA) approximation [18,19] can be applied. In this case the propagation of the quasi-monochromatic electromagnetic field of frequency ω along the *z*-axis

(2) $$\vec{E}(\vec{r},t)={\vec{E}}_{0}(\vec{r})\exp(i(kz-\omega t)),\;k=n_{0}\omega /c,$$

is reduced to the Schrödinger-type equation:

(3) $$ik\frac{\partial {\vec{E}}_{0}({\vec{r}}_{⊥},z)}{\partial z}=-\frac{1}{2}\nabla_{⊥}^{2}{\vec{E}}_{0}({\vec{r}}_{⊥},z)+\eta ({\vec{r}}_{⊥},z){\vec{E}}_{0},$$

where ${\vec{E}}_{0}$ is the slow-varying amplitude of the wave field, ${\vec{r}}_{⊥}=\left\{x,y\right\}$ are the coordinates perpendicular to the propagation direction, $\nabla_{⊥}^{2}$ is the Laplace operator over these coordinates and

(4) $$\eta ({\vec{r}}_{⊥},z)=-2\pi \;{\left(\omega /c\right)}^{2}\chi ({\vec{r}}_{⊥},z)$$

is the “potential” term that is defined by the susceptibility of the structure $\chi ({\vec{r}}_{⊥},z)$. The analogy between the wave Equation (3) and the Schrödinger equation is the core idea of the optical-mechanical analogy approach [17,20,21,22]. We remind that the media with χ>0 are known as focusing media and correspond to the attracting potentials in quantum mechanics. Otherwise, media with χ<0 are known as defocusing ones and correspond to the repulsive potentials in quantum mechanics. In Equation (3), the *z*-coordinate plays the same role as time in the usual non-stationary Schrodinger equation. From this point of view, the stationary beam profile propagating along the *z*-axis in nonhomogeneous media is quite similar to the particle wave function evolution in the two-dimensional potential. Further, we aim to study the planar structure when susceptibility depends only on one transverse coordinate x and the wave field is polarized along the y-direction.

In this work, the possible realization of an optical nonlinear device (“convertor”) mentioned in the introduction will be studied. The simplest geometry of such an optical system can be realized with the help of two planar waveguides that are coupled by a “tunnel” contact at a spatial length 𝓁 with each other. The energy fluxes of beams propagating in such a double-guide structure can be redistributed by controlling this “tunnel” contact. 

We suppose that the planar double-guide structure has the given susceptibility profile χ=χ(x,z) and the propagating beam is linearly polarized in the direction perpendicular to the x- and *z*-axes. Within the framework of the optical-mechanical analogy, such IP-element is similar to a pair of rectangular potential wells coupled through the potential barrier whose transparency could be varied in a controlled manner. By varying the barrier height one can “switch on/off” the tunneling probability of the particle to penetrate from one potential well to another. In optics, it means that for two waveguides with a rectangular form of susceptibility profile, one can organize the switching of signal from one channel to another and back via the control of the susceptibility in the between-waveguide-area at a certain length. Such a structure can be constructed on the base of semiconductor sheaths with different doping levels.

**Figure 1:**
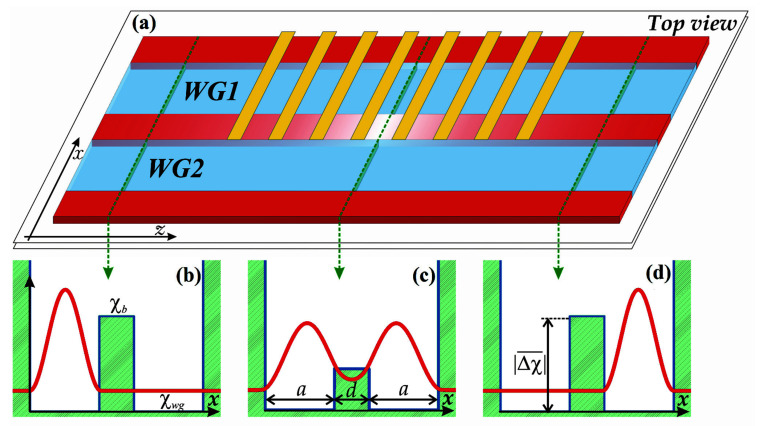
(**a**) The schematic top view of the two coupled planar waveguides (**a**) with additional control of the susceptibility in the middle part of the interwaveguide area (the yellow stripes illustrate the electrodes controlling the degree of doping in this area). In the framework of the optical-mechanical analogy, such a structure corresponds to the double-well potential (**b**–**d**). Plots (**b**,**d**) correspond to the switched-off tunnel transparency of the interwaveguide area, while the transparency is switched on for the plot (**c**). The red curves here are the snapshots of the initial/final field distribution (**b**,**d**) as well as field distribution during the tunneling (**c**).

To be more specific, let us consider the double waveguide structure with a rectangular susceptibility profile:

(5) $$\chi (x)=\left\{\begin{array}{l} \chi_{\infty },\;\left|x\right|\geq a+d/2,\\ \chi_{wg},\;d/2<\left|x\right|<a+d/2,\\ \chi_{b},\;\left|x\right|\leq d/2. \end{array}\right.$$

here a is the width of the waveguides, $\chi_{wg}$ and $\chi_{b}$ are susceptibilities of ones and interlayer between them respectively, d is the width of central barrier. Further, we will assume that we are able to change somehow the value of the susceptibility at a definite spatial interval. This change will give rise to the “tunnel” of the electromagnetic field from one guide to another (see Figure 1). Out of the structure $\left|x\right|\geq a+d/2$ we assume the permittivity $\varepsilon_{\infty }=1+4\pi \chi_{\infty }$ is strongly negative, so the electric field does not penetrate out of the waveguide to the surrounding medium. We suppose that the wave propagates along the waveguide (*z*-axis) with the wave vector.

(6) $$k=\left(\omega /c\right)\sqrt{1+4\pi \chi_{wg}},$$

Then the equation for the slow-varying amplitude $E_{0}(x,z)$ can be written in the form (3) with $\Delta \chi \left(x\right)=\chi \left(x\right)-\chi_{wg}$. If the susceptibility of the medium does not depend on *z* (such a situation corresponds to the stationary potential in quantum mechanics) the general solution of Equation (3) can be found as a decomposition over waveguide transverse eigenmodes:

(7) $$E_{0}(x,z)=\sum_{n}C_{n}X_{n}(x)\exp\left(-i\frac{\lambda_{n}^{2}}{2k}z\right),$$

where $X_{n}(x)$ and $\lambda_{n}$ [cm^−1^] obey the eigenvalue problem:

(8) $$\left(\frac{d^{2}}{dx^{2}}+4\pi {\left(\omega /c\right)}^{2}\Delta \chi (x)+\lambda_{n}^{2}\right)X_{n}(x)=0,$$

that is similar to the stationary Schrödinger equation.

The solution of Equation (8) gives rise to a number of transverse modes of the beam propagating in the *z*-direction. We use the following normalization condition for eigenfunctions $X_{n}(x)$:

(9) $$\int {\left|X_{n}(x)\right|}^{2}dx=\frac{{Ε}_{tot}^{2}}{8\pi }L.$$

Here, ${Ε}_{\mathrm{tot}}$ is the electric field strength in the mode and L=2a+d is the normalization length. All the eigenfunctions $X_{n}(x)$ are orthogonal to each other and the decomposition coefficients $C_{n}$ have the sense of population probability amplitude for the *n*-th transverse waveguide mode. The general solution (7) gives the spatial distribution of the electric field during the propagation along the *z*-axis. 

Let us analyze the structure of transverse modes in our double-waveguide structure. Inside each of the guides Δχ=0, in the space area between the guides ($\left|x\right|\leq d/2$) we have Δχ<0. Taking into account that the eigenfunctions $X_{n}(x)$ and their derivatives are continuous functions of the x coordinate, one obtains the following equation for the eigenvalue problem (8):

(10) $$\tan\;\lambda_{n}a=\frac{\lambda_{n}}{\lambda^{*}}\frac{1\pm \exp(-\lambda^{*}d)}{1\mp \exp(-\lambda^{*}d)}.$$

Here $\lambda^{*}=\sqrt{\Lambda^{2}-\lambda_{n}^{2}}>0$, $\Lambda^{2}=4\pi {\left(\omega /c\right)}^{2}\bar{\Delta \chi }$, $\bar{\Delta \chi }=\left|\chi_{b}-\chi_{wg}\right|$. The structure of the two lowest transverse modes is characterized by even and odd functions $E_{e}(x)$ and $E_{o}(x)$. As for the eigenvalues $\lambda_{1,2}=\lambda_{e,o}$, if the coupling of the waveguides is small, they can be found from Equation (10) in a form $\lambda_{e,o}\;a=\pi -\delta_{e,o}$ with

(11) $$\delta_{e,o}\approx \frac{\pi }{\Lambda a}\left(1\pm 2\exp(-\Lambda d)\right).$$

Here, index “e” corresponds to the even function and “o” to the odd one. The splitting of these modes is given by the expression

$$\Delta \lambda a=(\lambda_{o}-\lambda_{e})a\approx \frac{4\pi }{\Lambda a}\exp(-\Lambda d).$$

If coupling between the guides is almost absent, the even and odd modes are nearly degenerated and the eigenfunctions $E_{e}(x)$ and $E_{0}(x)$ are given by the expression:

(12) $$E_{e(o)}(x)\approx \sqrt{\frac{{Ε}_{tot}^{2}}{8\pi }}\left\{\begin{array}{l} \cos\left(\frac{\pi }{a}(x+d/2)\right),\;-a-d/2,<x<-d/2,\\ 0,\;\;\;\;\;\;\left|x\right|<d/2,\\ \pm \cos\left(\frac{\pi }{a}(x-d/2)\right),\;d/2<x<a+d/2. \end{array}\right.$$

Superposition of odd and even modes leads to the localization of the field in one of the waveguides (whereas in a quantum bit in a similar case we are dealing with the localization of the probability density distribution). For example,

(13) $$E(x)=\frac{1}{\sqrt{2}}(E_{o}(x)+E_{e}(x)).$$

corresponds to the wave propagating in the first guide (WG1), while

(14) $$E(x)=\frac{1}{\sqrt{2}}(E_{o}(x)-E_{e}(x)).$$

stands for the field propagation in the second guide (WG2).

The discussed mode splitting is similar to those in quantum theory when the two lowest states in the double-well potential with the tunnel coupling between them are split into even and odd states. The energy gap between these two states is governed by the coupling, i.e., by the probability to tunnel through the barrier between wells. If the particle is initially located in one of the wells it will transfer to the other well during the time interval that is inversely proportional to the energy gap ΔE between the odd state and the even one. The duration of this time interval $\tau^{*}\;$ is simply expressed through the value of energy splitting $\left(\Delta E/\hbar \right)\tau^{*}=\pi$.

A similar situation is realized in optics. “Tunneling” from one waveguide to another leads to the splitting of transverse modes. As a result, the energy flux transfer from one waveguide to another and back becomes possible upon the propagation of the signal in the structure. The length $L^{*}$ of transfer, i.e., the length at which the signal transfers from one waveguide to another, can be found in the equation

(15) $$\frac{\lambda_{o}^{2}-\lambda_{e}^{2}}{2k}L^{*}=\pi .$$

Taking into account the solution to the eigenvalue problem (10) one obtains

(16) $$L^{*}=ka^{2}/\left(\delta_{e}-\delta_{0}\right)\approx \frac{a}{4\pi }(ka)(\Lambda a)\exp(\Lambda d).$$

First, we note that the length $L^{*}$ of transfer exponentially depends on the parameter Λ. It means that by varying the permittivity of the internal “barrier” between the waveguides one can dramatically affect its transparency. This can be done for example by applying a static electric field to this region in order to influence the density of the electrons.

### 2.2. Estimates

Let us now perform some estimates. First, we suppose that the carrier wave frequency corresponds to the wavelength 1064 nm (the fundamental harmonic of the Nd laser). Regarding the material of the double waveguide structure, we analyze undoped GaAs for guide channels and n-doped GaAs with varying levels of doping for the barrier between them. The main parameters of the GaAs are given in Table 1. 

We use the following expression for the permittivity of an n-doped semiconductor:

(17) $$\varepsilon_{b}=\varepsilon_{0}-\omega_{p}^{2}/\omega^{2}.$$

Here, $\omega_{p}^{2}=4\pi e^{2}n_{e}/m^{*}$ is the plasma frequency squared and $n_{e}$ is the level of doping. We neglect the imaginary part in (17) as the transport frequency is significantly less than the carrier frequency of the radiation. First, as $\varepsilon_{b}=1+4\pi \chi_{b}$ and $\varepsilon_{0}=1+4\pi \chi_{wg}$, we find

(18) $$\Lambda^{2}=4\pi {\left(\omega /c\right)}^{2}\left|\chi_{b}-\chi_{wg}\right|={\left(\omega_{p}/c\right)}^{2}.$$

The inverse value $\delta =\Lambda^{-1}=c/\omega_{p}$ is known as a skin-depth and gives the estimate for the penetration depth of the radiation to the plasma medium. Hence, to effectively influence the tunnel transparency of the between waveguide barrier one needs the level of doping and the barrier width such that δ≥d .

If one chooses the double waveguide with a≈d≈1 mkm, the parameter is $ka=\sqrt{\varepsilon_{0}}(\omega /c)a\approx 20$. As for parameters $\Lambda a=\sqrt{\left|\varepsilon_{p}-\varepsilon_{0}\right|}(\omega /c)a\approx (\omega_{p}/c)a$ and $\Lambda d\approx (\omega_{p}/c)d$, they are approximately equal to each other and depend on the level of doping. If $\omega_{p}=2\times 10^{15}$ s^−1^ (such value of plasma frequency corresponds to the $8.5\times 10^{19}$ cm^−3^ level of GaAs doping), $\Lambda a\approx \Lambda d\approx (\omega_{p}/c)a\approx 6.7$. In this case, we have the length of transfer $L^{*}\approx 0.8$ cm.

## 3. Results and Discussion

**Figure 2:**
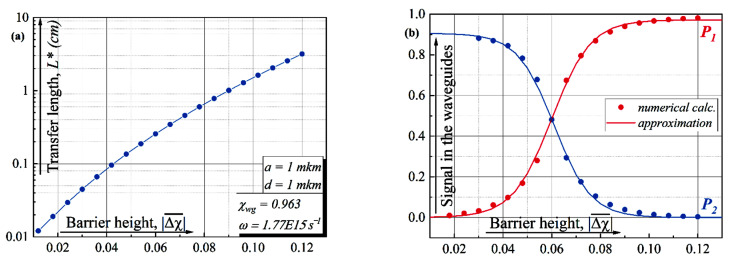
(**a**) The transfer length between the waveguides versus the “height of the barrier”. (**b**) The signal in the first/second waveguide (that is, the integral of the field energy in the corresponding region) versus the “height of the barrier”—dots, and the approximations of the results by sigmoidal functions—lines.

In this section, we will illustrate the application of the optical-mechanical analogy using the results of the numerical solution of Equation (3). We will start with the transformation of the electromagnetic signal from one waveguide to another in the considered structure for the case of varying “tunnel” transparency between the waveguides. From the point of view of a qubit state control, such an operation is equivalent to turning the state vector on the Bloch sphere around the *Ox* axis [25,26]. Let it assume, for example, that the wave packet at the input is localized in the first waveguide (the left potential well). In the framework of the optical-mechanical analogy, the wave function is a superposition of symmetric and antisymmetric eigenfunctions for the double-well potential (see Figure 1 and Equation (13)). The calculated lengths for the wave packet transfer between the waveguides are shown in Figure 2a for different fixed “heights” of the barrier between the waveguides. The process when the wave packet flows from one waveguide to another corresponds to the implementation of the “NOT” operation in a qubit.

Let’s assume that we are able to control the spatial interval 𝓁 at which we increase the transparency of the initially opaque spacer. Therefore, the signal in the first/second waveguide (that is, the integral of the field energy in the corresponding region) will depend on the controlled transparency of the interlayer. Hence, for given parameters, we can obtain a quasi-sigmoidal dependence of the signal intensity in the first/second waveguide on the interlayer susceptibility $\bar{\Delta \chi }\;$ during the smooth varying height of the barrier. Examples of such dependencies

$$P_{1}\sim \frac{0.972}{1+exp\left(-125\cdot \left(\bar{\Delta \chi }-0.06\right)\right)},$$

are shown in Figure 2b. We emphasize that these are typical activation functions for a neuron as part of an optical perceptron [27,28,29]. The advantage of the proposed concept is its conceptual simplicity, while the disadvantage is the use of electronic control to change the transparency of the barrier between waveguides.

Everywhere before in analogy with our analytical model, we assumed the sharp switch-on profile of the interlayer waveguide susceptibility along the *z*-direction at a spatial point z=0. The numerical solution of the optical Schrodinger Equation (3) makes it possible to investigate the behavior of the system in practically more important cases when the susceptibility of the waveguide interlayer material changes smoothly in space along the propagation direction, z. For certainty, consider the change in the initial susceptibility of the barrier $\bar{\Delta \chi \left(z\right)}$ along the *z*-direction in the form of a smoothed trapezoid:

(19) $$\bar{\Delta \chi \left(z\right)}=-C\left(\frac{1}{1+exp\left(-D\cdot \left(z-z_{r0}\right)\right)}+\frac{1}{1+exp\left(+D\cdot \left(z-z_{f0}\right)\right)}-1\right),$$

where the parameter *D* is responsible for the smoothness of Δχ profile. Below, for clarity, we will use the rise/fall distance, *z_r/f_*, which depends on *D* (for *D* = 4673 cm^−1^ $z_{r}=z_{f}=0.046$ mm). The values of *z_r_*_0_ and *z_f_*_0_ determine the beginning and end of the region where it is possible for the field to flow from one waveguide to another, $𝓁\approx \left|z_{r0}-z_{f0}\right|$ is the length of the coupling region. We note that chosen below values *z_r/f_* are significantly less than the typical values of transfer length (see Figure 2a). Hence, the further data should be consistent with the analytical model from Section 2. 

**Figure 3:**
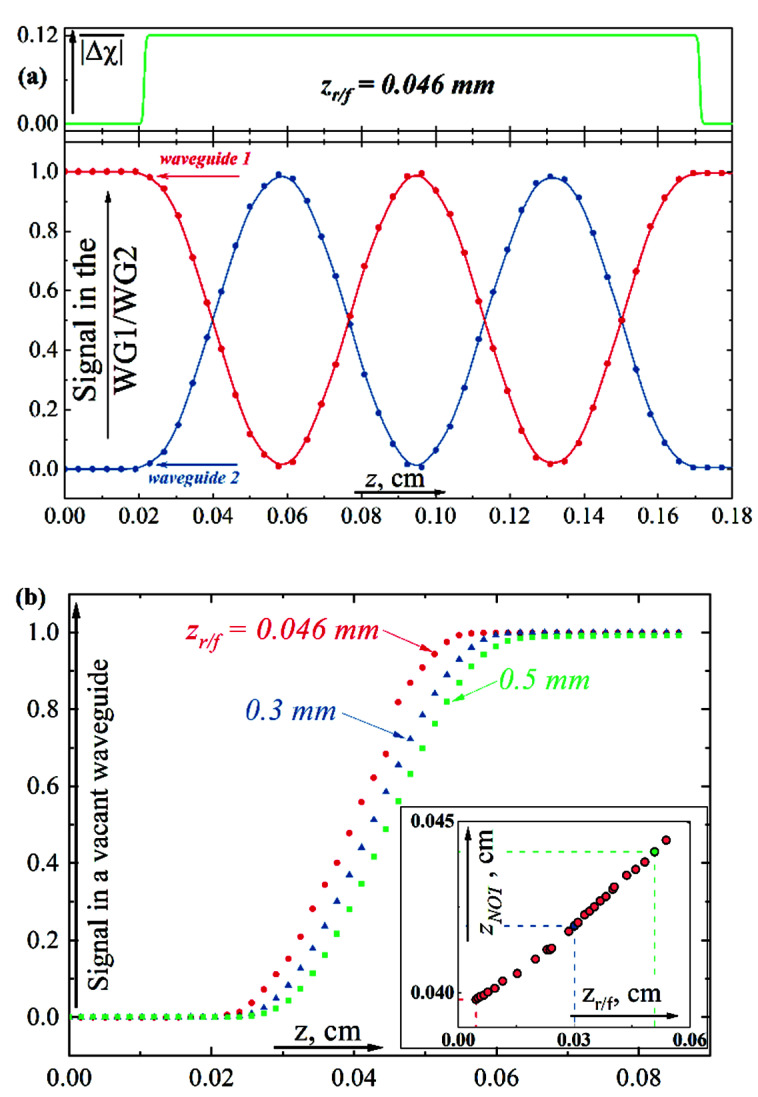
(**a**) The signal in the first/second waveguide versus *z*-coordinate for the smoothed function $\Delta \chi \left(z\right)$, *z_r/f_* ≈ 0.046 mm. The upper part of the figure presents a decrease in the “height” of the barrier separating the waveguides (minima of the double-well potential) along the *z*-axes. (**b**) The signal in a vacant waveguide versus *z*-coordinate for the NOT-operation. The inset shows how the spatial size required for the NOT operation increases with the degree of smoothing $\Delta \chi \left(z\right)$.

The dynamics of signal transfer from one waveguide to another during its propagation along the z-direction is clearly demonstrated in Figure 3a. We suppose that initially the electromagnetic signal is located in the first guide. For the given parameters we observe the transfer of electromagnetic energy from one guide to another and back (see Figure 3a). This process is quite similar to oscillations of the probability density in two-level quantum systems with a tunnel coupling between the wells [13]. For clarity, in the same figure, we have deduced a decrease in the “height” of the barrier separating the waveguides (minima of the double-well potential) along the z-axes.

Once again, here we can implement an analog of the “NOT” operation on the “qubit” state in the photonics domain varying *z_r/f_* parameters. In Figure 3b, we present the dependence of the signal in the vacant waveguide (WG2) as a function of the *z*-coordinate for the implementation of a NOT-operation in the classical optical analog of a solid-state qubit for three values *z_r/f_*. It can be seen from the inset to the figure that the *z*-size of the waveguide—*z_NOT_*—required for such a process increases linearly with the degree of smoothing of the function.

Another possible way to realize the optical “convertor” is to control the symmetry of the double-well potential by changing the depth of one of the wells. In optics, it means that the susceptibility of one of the waveguides is changed along the direction of the electromagnetic signal propagation. The asymmetry characterizes the difference between the susceptibilities of the waveguides. From the point of view of a qubit state control, such an operation is equivalent to turning the state vector on the Bloch sphere around the *Oz* axis. However, an adequate analysis of this problem requires further research.

**Figure 4:**
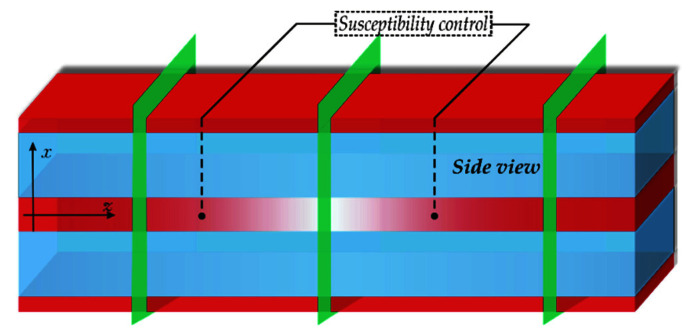
The schematic side view of the two coupled planar waveguides with additional control of the susceptibility in the middle part in a three-dimensional topology. Susceptibility of one of them is changed along the direction of the signal propagation.

The transition to a three-dimensional topology (as shown in Figure 4) will increase the degree of integration of schemes containing the considered IP-element. This is very important for modern quantum and classical optics on a chip [30,31,32]. The advantages of such schemes are the possibility of scaling and reprogramming. However, today a number of quantum information protocols are also used by “atomic systems”, where the methods for recording, storing and reading quantum information are easily implemented. Our study, where a pair of waveguides connected by resonant tunneling acts as an analog of the “atomic system”, was carried out in the spirit of these investigations.

Here we would like to draw your attention to the fact that a nonlinear activation function for optical neurons appears in a model based on a linear wave equation of the Schrödinger type. This is because the tunneling probability is characterized by a strongly nonlinear dependence on the parameters of the barrier. Moreover, the specifics of tunneling through a potential barrier in a quantum system with a double-well potential (a double quantum dot; a flux qubit) gives a spectrum where the ground and first excited states are separated by a relatively large energy gap from the next energy levels [33,34,35]. Such a spectrum is convenient for the implementation of artificial two-level quantum systems. In our case, an analog of such a system can be implemented in a completely classical system. The optical-mechanical analogy helped us to understand that changing the height of the barrier and the asymmetry gives a complete set of the single-qubit operations in the system (a set of *Z*-and *X*-rotations, respectively) [13].

In the summary, let us emphasize the new results of the research. We have determined, for the first time, the parameters at which a pair of waveguides can play the role of a classical analog of solid-state qubits. In the future, the approach tested here will allow us to reproduce in classical optical schemes a number of important physical effects known in atomic quantum optics and quantum computer science: Rabi or Ramsey oscillations, Landau-Zener transition, etc. [36]. Using the proposed test structure, one can test the interaction of qubits and qutrites with super-strong and ultrashort control pulses [37,38,39] without the use of expensive and sophisticated cryogenic sensors and other equipment. We also showed for the first time that a pair of waveguides under certain conditions can be used as an optical neuron with a sigmoidal activation function.

## Figures and Tables

**Table 1:** Main parameters of GaAs semiconductor [23,24].

Material	Effective Mass $m^{*}$ (in Masses of Free Electron)	Forbidden Zone, eV	Permittivity of the Undoped Material, $\varepsilon_{0}$	Electron Mobility, cm^2^/V-s	Transport Frequency, s^−1^
GaAs	0.067	1.424	13	8500	$3\times 10^{12}$
